# Modic changes of the lumbar spine—their association with the lumbar sagittal parameters: A retrospective imaging study

**DOI:** 10.1186/s13018-020-01745-z

**Published:** 2020-06-15

**Authors:** Xiaoping Mu, Wei Peng, Chengqiang Yu, Jian Xiong, Jianxun Wei, Yufu Ou, Chenglong Wang

**Affiliations:** 1 Department of Orthopedics, People’s Hospital of Guangxi Zhuang Autonomous Region, Nanning, 530021 China; 2 Institute of Anatomy and Cell Biology, Justus-Liebig-University, 35392 Giessen, Germany; 3 Faculty of Acupuncture, Guangxi University of Chinese Medicine, Nanning, 530001 China

**Keywords:** Lumbar spine, Vertebral endplate signal changes, Modic changes, Lumbar sagittal parameters, Correlative factors, Imaging study

## Abstract

**Background:**

The Lumbar sagittal parameters might be related to modic changes (MCs). However, studies on this topic have rarely been reported. The aim of this study was to identify the relationships between the lumbar sagittal parameters and the development of MCs.

**Methods:**

The lumbar sagittal parameters of 321 patients with chronic low back pain from May 2016 to August 2018 were measured on X-ray by using Surgimap surgical planning software. Univariable analyses were used to test the potential variables of interest. Logistic regression models were then performed for the significant parameters to identify the independent factors associated with the development of MCs.

**Results:**

More patients in the MCs group were older with more number of female than in the disc degeneration group (*p* < 0.05). In the univariate analysis, significant differences were detected for the parameters of lumbar lordosis, sacral slope, intervertebral height index, endplate concave angle, and intervertebral angle only at the L5/S1 level between the two groups. The results of logistic regression analysis showed that a smaller intervertebral height index was positively associated with the development of MCs at the level of L3/4 (*p* < 0.05). However, the positive role of gender was only for MCs at the L5/S1 level (*p* < 0.05).

**Conclusions:**

The results of this study revealed that there were negative relationships between the lumbar sagittal parameters and MCs. Furthermore, being female and having a narrow intervertebral space were the independent risk factors for the development of MCs at the corresponding lumbar levels. Interestingly, body mass index might be not associated with MCs for the Chinese population.

## Background

Vertebral endplate subchondral bone signal changes, also known as modic changes (MCs), are detected clearly on magnetic resonance imaging (MRI). de Roos et al. [1] initially reported on the abnormal signal changes in the vertebral body marrow adjacent to the endplates in the patients with lumbar disc degeneration. Subsequently, Modic et al. [2, 3] described in detail their histological features and the three types of MCs based on their appearance on T1-weighted and T2-weighted images in 1988.

In recent years, more attention has been paid to MCs, which are positively associated with chronic low back pain (CLBP) [4, 5]. Although MCs have been studied for several decades, the potential pain mechanisms are still unclear or controversial. Endplate inflammation or low-toxicity infection may be the cause of pain for patients with MCs [6–8]. However, several studies have shown that spinal instability also plays an important role in the occurrence of pain [9, 10].

Spine stability is determined by the shape of the spine, composed of several normal spinal motor units. Moreover, the shape of the spine can be reflected by sagittal balance, which allows humans to maintain the standing position with little muscle effort [11]. Previous studies have suggested that sagittal balance status, as an independent predictor, not only affects the clinical symptoms of many spinal diseases, but also plays a positive role in the prognosis of patients who underwent appropriate treatment [12, 13]. In addition, studies of sagittal parameters in MCs of the cervical spine showed that the T1 slope [14] and the C2–C7 Cobb angle [15] are potential risk factors for the development of MCs.

Lumbar sagittal parameters may also be related to the development of MCs, but which ones have strongly associations with MCs remain unpredictable. To the best of our knowledge, data on the correlation between MCs and sagittal parameters in the lumbar spine are limited in the literature. Therefore, the main purpose of this study was to identify the relationships between lumbar sagittal parameters and development of MCs.

## Materials and methods

### Patients population

In this retrospective study, the sample cohort was derived from 1019 consecutive patients with lumbar disc degenerative diseases referred for a standard lumbar X-ray (standing position) and MRI (supine position) from May 2016 to August 2018 in our clinic. Most patients had a history of low back pain and their symptoms met the diagnostic criteria for non-specific CLBP [16, 17]. The duration of symptoms ranged from 3.8 to 8.7 months. In order to avoid the impact of previous treatments, only those patients who initially sought medical help for CLBP and did not receive any prior treatment were included. In addition, only adult patients whose disc degeneration (DD) grade was rated as grade 3 or greater in the Pfirrmann classification were included. The exclusion criteria included a history of lumbar surgery, spinal deformities, spinal infection, tumor, rheumatoid arthritis, and abnormal gait and posture caused by disability of the lower extremity or other diseases.

**Fig. 1:**
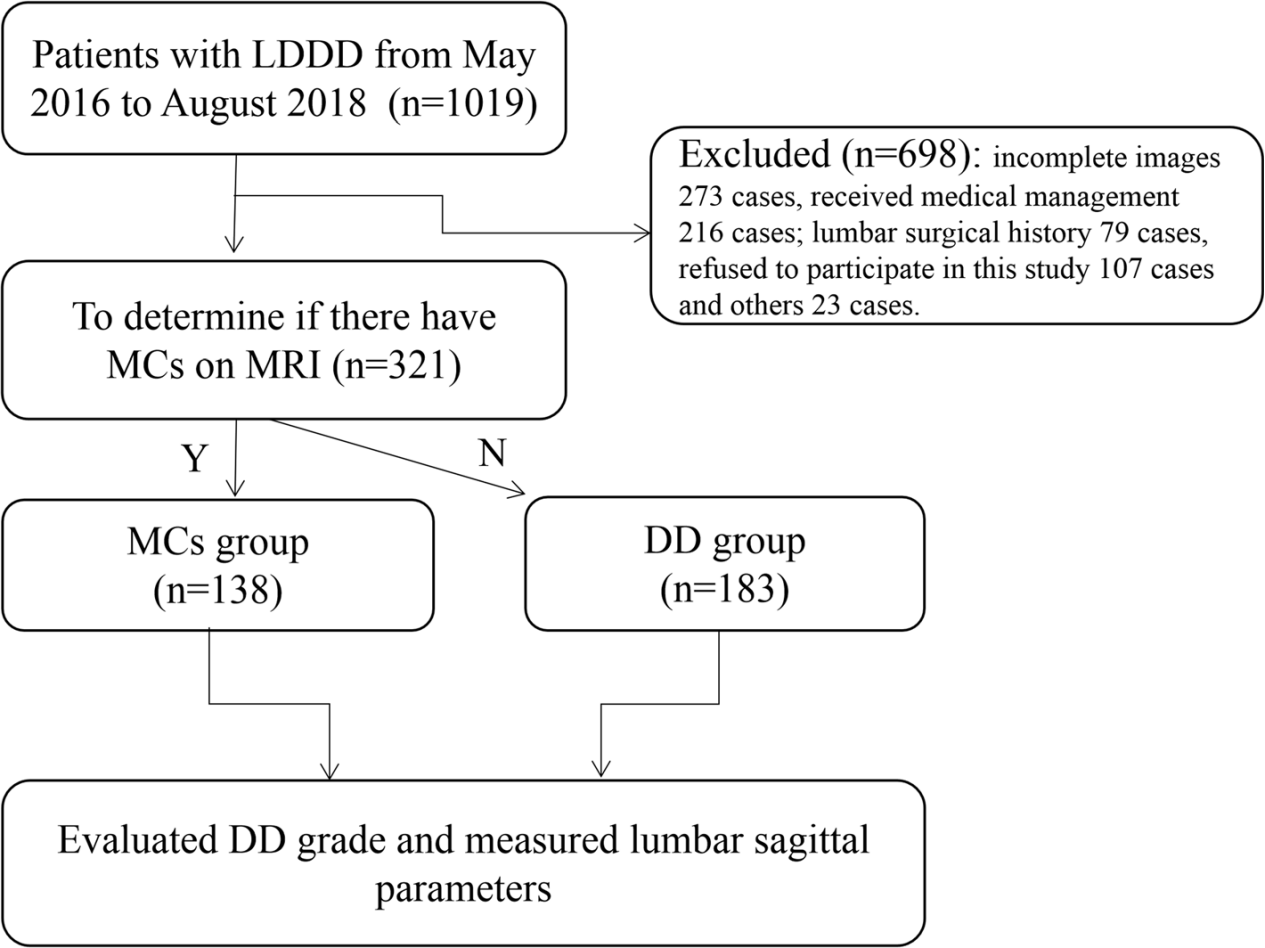
The selection process of study population. LDDD: lumbar degenerative disc disease; MRI: magnetic resonance imaging; MCs: Modic changes; DD: disc degeneration

Finally, we identified 321 patients. Of which, 138 patients (81 females and 57 males) had MCs, with a mean age of 52.64 years, were included in the MCs group. Considering that DD has been shown to be closely associated with MCs [4, 5] and that lumbar segmental stability has been determined for both the lumbar disc and the endplate [9], the remaining 183 patients without MCs (73 females and 110 males; mean age, 48.99 years), with grade 3 or greater of DD, were allocated to the DD group. The study population selection process is shown in Fig. 1.

All patients allowed us to review their medical records and signed the relevant informed consent. The study protocol was approved by the Institutional Review Board of the People’s Hospital of Guangxi Zhuang Autonomous Region (no. 2016-12). This clinical investigation was conducted in strict accordance with the principles of the Declaration of Helsinki.

### Imaging evaluation and measurement

The lumbar X-ray (standing position) and MRI (supine position) examination were performed in accordance with the standard scanning procedure. Two experienced observers (M.X.P and W.C.L) evaluated and measured all patients images blinded to the patients demographics and clinical profiles by using the Surgimap surgical planning software (version 2.2.15, Globus Medical, Inc., Audubon, PA, USA).

Based on the classification system proposed by Modic et al. [2, 3], all MCs were divided into three categories from T1- and T2-weighted images: type I, hypointense on T1-weighted and hyperintense on T2-weighted images; type II, hyperintense both on T1- and T2-weighted images; and type III, hypointense on both T1- and T2-weighted images. Based on the grading method proposed by Pfirrmann [18], DD was classified into five grades from T2-weighted imaging (Table 1).

**Table 1:** Pfirrmann grading system of lumbar disc degeneration

Grade	Structure	Distinction of nucleus and annulus	Signal intensity	Disc height
I	Homogeneous, bright white	Clear	Hyperintense, isointense to CSF	Normal
II	Inhomogeneous with or without horizontal bands	Clear	Hyperintense, isointense to CSF	Normal
III	Inhomogeneous, gray	Unclear	Intermediate to CSF	Normal to slightly decreased
IV	Inhomogeneous, gray to black	Lost	Intermediate to hypointense to CSF	Normal to moderately decreased
V	Inhomogeneous black	Lost	Hypointense to CSF	Collapsed

*CSF* cerebrospinal fluid

**Fig. 2:**
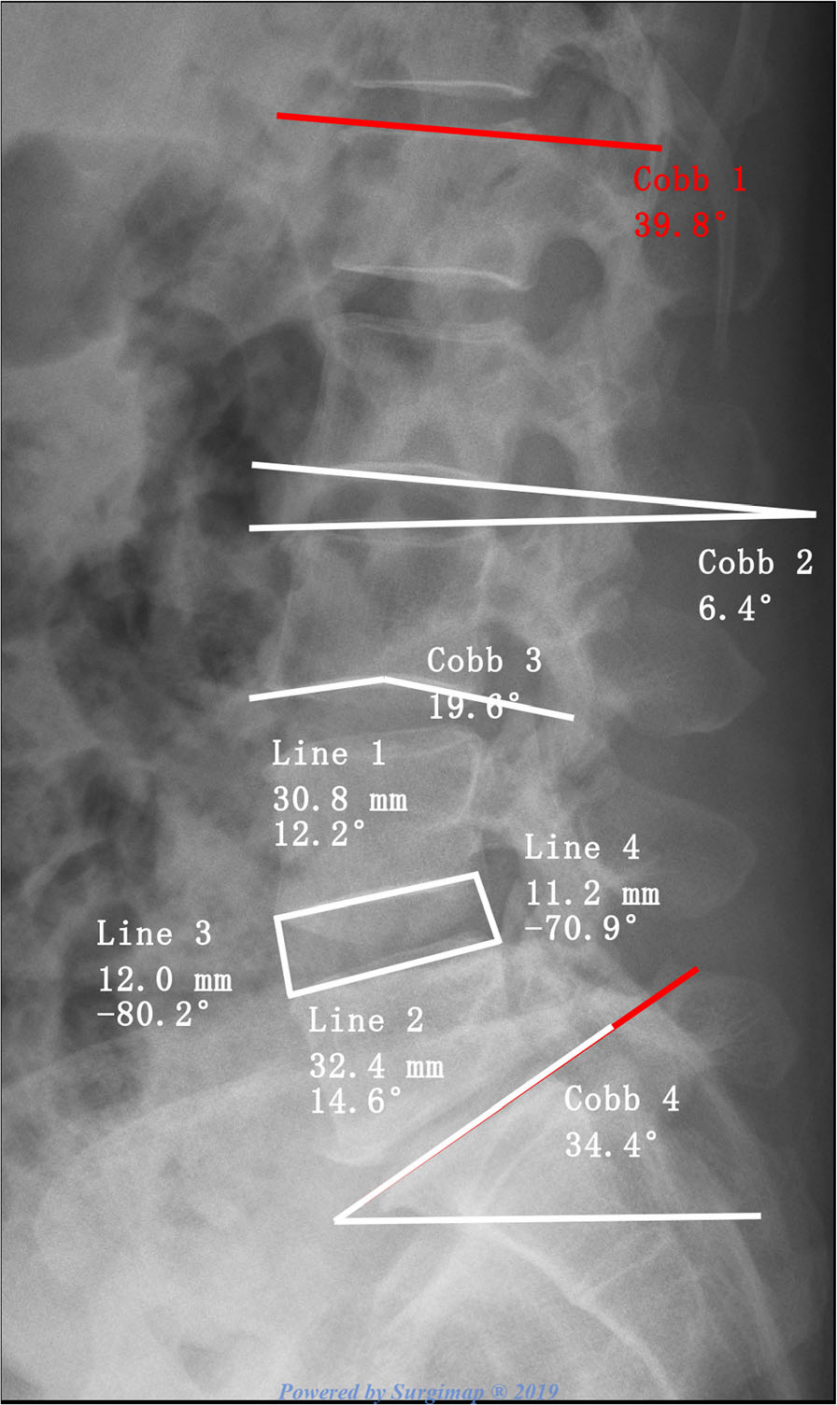
Measurements of lumbar lordosis (LL), sacral slope (SS), intervertebral height index (IHI), intervertebral angle (IVA), and endplate concave angle (ECA). Cobb 1(red): LL; Cobb 2: IVA; Cobb 3: cranial ECA; Cobb 4: SS; IHI = (line 1 + line 2)/(line 3 + line 4) × 100%

Lumbar lordosis (LL) was defined as an angle formed by two oblique lines through and parallel to the L1 and S1 cranial endplates, respectively. Sacral slope (SS) was defined as an angle between a horizontal line and the cranial endplate of S1. The endplate concave angle (ECA) was measured as an angle formed by the lines drawn from the bottom or summit of the arc along to their endpoints. The intervertebral height index (IHI) = (anterior disc height + posterior disc height)/(superior disc diameter + inferior disc diameter) × 100%. The intervertebral angle (IVA) was measured as the angle of two lines through and parallel to the cranial and caudal endplates (Fig. 2).

### Statistical analysis

IBM SPSS Statistic software (Version 21.0; IBM Corp., Armonk, NY, USA) was used for the statistical analyses. The main manifestations of the data results are presented as the mean±standard deviation (SD). Appropriate statistical methods (Mann-Whitney *U* test or *t* test for measurement data, and the *χ*^*2*^ test for count data) were performed to detect the difference between the MCs and DD groups. The potential variables of interest were tested first. Subsequently, logistic regression models were performed for the significant parameters to identify the independent factors associated with the development of MCs. The intraclass correlation coefficient (ICC) was used to evaluated the inter-observer and intra-observer variability bias. Statistically significant differences were defined as those having a two-tailed *p* value < 0.05.

## Results

All of the X-ray and MRI images were reviewed in accordance with the study protocol. Finally, 536 lumbar segments with grade 3 or greater of DD (MCs group: 158 lumbar segments from 138 patients; DD group: 378 lumbar segments from 183 patients) were measured for the sagittal parameters on lumbar X-ray images. Due to a lack of sufficient samples (MCs group at L1/2: 1 cases; MCs group at L2/3: 3 cases), the parameters of L1/2 and L2/3 could not be analyzed in this study.

### Prevalence of MCs and patient demographics

Overall, 138 patients had MCs (42.99%). The most common distribution of MCs was at the L5/S1 (53.80%) and L4/L5 (32.91%) level, with type II (69.57%) being the most common. The incidence of MCs in females (58.70%) was higher than in males (41.30%). The characteristics of patients in these two groups are shown in Table 2. No significant differences were found in the body mass index (BMI), white blood cell count (WBC), and the C-reactive protein (CRP) level between the two groups (*p* > 0.05). However, older age and female sex were the most common characteristics in patients with MCs compared with patients in the DD group (*p* < 0.05).

**Table 2:** The patients’ demographics

Items	MCs group (*n* = 138)	DD group (*n* = 183)	*z*/*x*^2^	*p*
Age (years)	52.64 ± 11.69	48.99 ± 13.66	2.579	0.010
Gender (m/f)	57/81	110/73	11.147	0.001
BMI	22.80 ± 2.85	22.70 ± 2.50	0.348	0.728
WBC (+)	8.11 ± 2.79	7.74 ± 2.54	− 1.051	0.293
CRP (+)	8.65 ± 17.18	7.01 ± 9.07	− 0.483	0.629

### Inter-observer and intra-observer reliability

Excellent agreement was observed between the reviewers for the classification of MCs (ICC value 0.94), their types (ICC value 0.87), and grade of DD (ICC value 0.86). There was the substantial agreement between the two reviewers on the assessment of LL (ICC value 0.79), SS (ICC value 0.71), and IVA (ICC value 0.73). Two reviewers had moderate agreement on the ECA (ICC value 0.59) and IHI (ICC value 0.51). There were excellent intra-observer agreement on the MCs classification (observer 1 0.98 and observer 2 0.99), grade of DD (observer 1 0.87 and observer 2 0.91), the LL (observer 1 0.85 and observer 2 0.83), SS (observer 1 0.82 and observer 2 0.87), IVA (observer 1 0.88 and observer 2 0.83), IHI (observer 1 0.85 and observer 2: 0.82), and ECA (observer 1 0.81 and observer 2 0.84).

### Independent risk factors

Potential variables of interest of the MCs and DD groups are compared in Table 3. In the univariate analysis, significant differences were detected between the MCs and DD groups for the parameters of LL, SS, IHI, ECA, and IVA only at the L5/S1 level. Furthermore, multivariate logistic regression was performed to evaluate the potential factors related to MCs.

**Table 3:** The results of the potential variables of interest

Items		MCs group (*n* = 138)	DD group (*n* = 183)	*Z*	*p*
LL (°)		33.77 ± 11.52	39.22 ± 11.53	− 4.192	0.000
SS (°)		31.55 ± 8.40	35.20 ± 7.81	− 4.007	0.000
IHI	L3/4	23.32 ± 5.05	30.37 ± 4.17	− 5.124	0.000
	L4/5	27.30 ± 6.65	32.44 ± 5.33	− 5.197	0.000
	L5/S1	30.35 ± 6.98	34.18 ± 5.98	− 4.413	0.000
IVA (°)	L3/4	6.14 ± 2.80	6.10 ± 2.61	− 0.258	0.796
	L4/5	6.61 ± 3.58	6.85 ± 3.80	− 0.424	0.671
	L5/S1	10.11 ± 5.43	12.30 ± 4.87	− 2.853	0.004
ECA (°)	L3/4 U	159.19 ± 7.61	164.68 ± 10.34	− 3.127	0.002
	L3/4 L	162.45 ± 13.78	169.98 ± 6.67	− 2.318	0.020
	L4/5 U	160.93 ± 10.79	165.62 ± 8.40	− 2.852	0.004
	L4/5 L	166.06 ± 9.39	171.63 ± 6.45	− 3.775	0.000
	L5/S1 U	157.39 ± 11.29	162.23 ± 8.02	− 3.616	0.000
	L5/S1 L	167.36 ± 9.22	171.33 ± 7.09	− 3.894	0.000

To avoid the effect of lumbar levels on the results of the study, we performed a separate logistic regression analysis for each lumbar level. The results of logistic regression analysis showed no significant relationships between age, LL, SS, ECA, IVA, and the development of MCs. A reduction in IHI only at the L3/4 level was associated with the presence of MCs (odds ratio [OR] 7.77; 95% confidence interval [CI] 2.037–29.614; *p* = 0.003). A 7.77-fold increased risk was found for the presence of any MCs, with every 1% reduction in the IHI for the lumbar spine. For a female at the L5/S1 level there was an association with an increased risk of the presence of MCs (OR 2.33; 95% CI 1.247–4.361; *p* = 0.008) (Table 4).

**Table 4:** The logistic regression analysis of potential factors related to MCs

Risk factors	β	SE	Wald	*p*	OR	95% CI
L3/4						
IHI	2.050	0.683	9.010	0.003	7.767	2.037~29.614
L5/S1						
Gender	0.847	0.319	7.022	0.008	2.332	1.247~4.361

## Discussion

### Main findings

The lumbar instability caused by spinal degenerative changes may be an important factor for the development of MCs. However, no previous studies were designed to test the possible relationships. To the best of our knowledge, this is the first study to report on the relationships between multiple sagittal parameters of the lumbar spine and the development of MCs. The results of this study showed negative relationships between the lumbar sagittal parameters and MCs. Furthermore, female sex and a narrow intervertebral space were independent risk factors for the development of MCs at the corresponding lumbar levels. This study also reinforces the strength of the findings from previous studies which have reported that MCs have a high incidence among patients with DD. Interestingly, our study also indicated that BMI might not be associated with MCs in the Chinese population.

### Prevalence of MCs

Previous studies have reported that the incidence of MCs among patients with lumbar DD varies between 19% and 59%, with type 2 being the most common and L5/S1 being the most common level [1, 9, 19–22]. Our findings were consistent with those of the earlier studies with the conclusions that MCs have a higher incidence among patients with lumbar DD (42.99%), their prevalence increased with age, and female comprise the majority of MCs cases, with type 2 and L5/S1 being the most common. Some studies [4, 23] have reported that the prevalence of MCs among non-clinical populations in Asia was much lower than that in Europe and North America. However, our study indicates that the prevalence rates of MCs were not lower than those in other ethnicities, which may be attributed to the fact that the subjects being recruited in this study were selected DD cases. This study also shows that female sex is a determinant of MCs. Female sex also increased the odds of MCs at the L5/S1 level by 2.3-fold. The high incidence of osteoporosis in females may be one of the reasonable explanations for the high prevalence of MCs in females [24].

In our study, age is associated with an increased risk of MCs among patients with DD, but BMI, CRP, and WBC did not increase the likelihood of MCs. Age and BMI have previously been proven to be related to the occurrence of MCs [5, 19]. Interestingly, our study showed no significant differences in BMI between the two groups. A recent population-based study [4] conducted with a Chinese population reported that no significant differences were found in BMI. A plausible explanation may be due to the BMI not increasing the likelihood of MCs in the Chinese population. MCs have been proven to be strongly associated with CLBP [20]. Park et al. [25] reported that CRP is not a predictor in patients with CLBP, and our findings are consistent with those of the earlier studies.

### Lumbar sagittal parameters

LL is unique to the human spine and develops an upright posture as facilitation progresses, and loss of LL causes irregular stress distribution on the spine [26]. SS is used to evaluate the normal variation in the sagittal plane of the adult human lumbar spine and pelvis in the standing position [27]. A recent study reported by Xia et al. [28] supports that the amount of MCs was significantly correlated with LL and SS. This is coincident with our study. The associations of LL and SS with risk of MCs may be due to decreased axial decompression ability of the spine and an increased shear force on the vertebral endplate [28]. This would increase the risk of endplate and cancellous bone damage when the spine is subjected to external forces in the perpendicular direction [29].

The IHI, as a quantitative and continuous measure, and the relationships between IHI and DD, were explored in a study by Teichtahl [30] that supported the idea that a negative dose–response relationship exists between the increasing severity of DD and a reduction of the IHI. For every 1 mm reduction in the total intervertebral disc height for the lumbar spine, there was a 1.1-fold increased risk for the presence of MCs, which was described by Teichtahl [31]. In the present study, we observed that the IHI is significantly associated with an increased risk of MCs, which may be due to a decreased IHI attenuation of the cushioning effect and load redistribution to adjacent vertebrae, resulting in MCs [31]. In the multivariate logistic regression, after adjusting for age, WBC, BMI, and CRP, a reduction in the IHI at the L3/L4 level was associated with the presence of MCs. There was a 7.77-fold increased risk for the presence of any MCs with every 1% reduction in the IHI for the lumbar spine.

Endplate concavity is conducive to humans for long-term adaptation to pressure stress and dispersion of axial stress [32]. The present study supports a negative correlation between MCs and the vertebral ECA. A previous study [32] reporting on the associations of the ECA with severity of DD indicated that as the ECA increased, with the endplate becoming flat, closely correlated with the severity of the DD. However, this study did not report that the ECA is associated with MCs. Li et al. [33] demonstrated that the incidence of MCs increased with the change in sagittal endplates from concave to flat to irregular and indicated that an irregular shape of the endplate is significantly associated with MCs. It is important to note that the ECA will be deceased with the change in sagittal endplates from flat to irregular. Our finding is consistent with the findings of Li et al., which may be due to the endplate remodeling involved in the pathogenesis of MCs and can be considered a response to abnormal segmental mobility [33]. However, compared with the shape of the endplate that was used to designate the level as concave, flat, and irregular, the ECA is a more precise measure of the shape of the endplate.

In addition, our study indicates that significant differences were detected between the MCs and DD groups for IVA, but only at the L5/S1 level. Hayashi et al. [34] reported that a significant decrease of angular motion was found in segments with MCs in the severe DD stage. Their study was consistent with our finding because decreased or collapsed disc height due to DD would result in decreased IVA and angular motion.

### Limitations

The present study has several limitations. One major drawback of this study is the retrospective nature of this study which makes it difficult to control some variables inherent for patients with MCs. Importantly, a lack of whole spine radiographs forced us to abandon the measurement and analysis of pelvic parameters due to the cost to the patient and local medical insurance policies. In addition, the development of MCs has been well known to be a dynamic process, and lumbar sagittal parameters may be affected by different types of MCs. Thus, another problem of this study is that it did not consider all types of MCs because of a limitation of the sample size. Although complete elimination of the measurement error is impossible, we attempted to keep the measurement error within acceptable tolerance. Finally, the possibility of selection bias exists in this study.

## Conclusions

In conclusion, DD and lumbar level should be considered in evaluating the relationships between lumbar sagittal parameters and the development of MCs. This study reinforces the strength of the findings from previous studies, which reported that MCs have a high incidence among patients with DD. Interestingly, it also indicated that BMI might be not associated with MCs for the Chinese population. The results of lumbar sagittal parameters for patients in the MCs group were worse than those in the DD group, indicating that lumbar sagittal parameters were negatively associated with the development of MCs. Furthermore, the results of logistic regression analysis suggested that the narrow intervertebral space was the independent risk factor for the development of MCs at the L3/L4 level. However, the positive role of sex was only found for MCs at the L5/S1 level.

## Data Availability

The datasets generated and analyzed during the current study are available from the corresponding author on reasonable request.

## Abbreviations

MCs: Modic changes
MRI: Magnetic resonance imaging
CLBP: Chronic low back pain
DD: Disc degeneration
LL: Lumbar lordosis;
SS: Sacral slope
IHI: Intervertebral height index
ECA: Endplate concave angle
IVA: Intervertebral angle
